# Complete agreement of the post-spinel transition with the 660-km seismic discontinuity

**DOI:** 10.1038/s41598-018-24832-y

**Published:** 2018-04-20

**Authors:** Takayuki Ishii, Rong Huang, Hongzhan Fei, Iuliia Koemets, Zhaodong Liu, Fumiya Maeda, Liang Yuan, Lin Wang, Dmitry Druzhbin, Takafumi Yamamoto, Shrikant Bhat, Robert Farla, Takaaki Kawazoe, Noriyoshi Tsujino, Eleonora Kulik, Yuji Higo, Yoshinori Tange, Tomoo Katsura

**Affiliations:** 1 Bayerisches Geoinstitut, University of Bayreuth, 95440 Bayreuth, Germany; 2 Department of Earth Sciences, Graduate School of Science, Tohoku University, Sendai, 980-0845 Japan; 3 Department of Earth and Planetary Systems Sciences, Graduate School of Science, Hiroshima University, Kagamiyama 1-3-1, Higashi-Hiroshima, 739-8526 Japan; 4 Deutsche Elektronen-Synchrotron (DESY), Notkestraße 85, 22607 Hamburg, Germany; 5 Institute for Study of the Earth’s Interior, Okayama University, Misasa, 682-0193 Japan; 6 Japan Synchrotron Radiation Research Institute (JASRI), 1-1-1, Kouto, Sayo-cho, Sayo-gun, Hyogo, 679-5198 Japan

## Abstract

The 660-km seismic discontinuity, which is a significant structure in the Earth’s mantle, is generally interpreted as the post-spinel transition, as indicated by the decomposition of ringwoodite to bridgmanite + ferropericlase. All precise high-pressure and high-temperature experiments nevertheless report 0.5–2 GPa lower transition pressures than those expected at the discontinuity depth (i.e. 23.4 GPa). These results are inconsistent with the post-spinel transition hypothesis and, therefore, do not support widely accepted models of mantle composition such as the pyrolite and CI chondrite models. Here, we present new experimental data showing post-spinel transition pressures in complete agreement with the 660-km discontinuity depth obtained by high-resolution *in situ* X-ray diffraction in a large-volume high-pressure apparatus with a tightly controlled sample pressure. These data affirm the applicability of the prevailing mantle models. We infer that the apparently lower pressures reported by previous studies are experimental artefacts due to the pressure drop upon heating. The present results indicate the necessity of reinvestigating the position of mantle mineral phase boundaries previously obtained by *in situ* X-ray diffraction in high-pressure–temperature apparatuses.

## Introduction

The 660-km seismic discontinuity divides the upper and lower mantle. It is one of the most important boundaries in the Earth’s interior and controls a number of the mantle’s dynamic processes, such as slab stagnation. In the widely accepted models of the composition of the mantle (e.g. the pyrolite and CI chondrite models), the major constituent of the lower part of the upper mantle (the transition zone) is (Mg,Fe)_2_SiO_4_ in the form of ringwoodite, which decomposes into bridgmanite + (ferro) periclase—the post-spinel transition—at a pressure near that at the 660-km discontinuity (23.4 GPa)^1–5^. Therefore, the post-spinel transition is commonly accepted to be the cause of the 660-km discontinuity. Beginning in the late 1990s, the post-spinel transition pressure in Mg_2_SiO_4_ has been investigated using a combination of large volume presses (LVPs) and synchrotron-based *in situ* X-ray diffraction^2–5^, which enabled the most precise and accurate determination of the phase boundary^6^. The near absence of an iron effect on the transition pressure^1^ justifies the use of Mg_2_SiO_4_ samples. Nevertheless, these studies have located the post-spinel transition at pressures of 21.4–22.9 GPa at temperatures of 1900–2000 K (the geotherm at a depth of 660 km^7,8^), which are 0.5–2.0 GPa lower than the actual pressure at a depth of 660 km. If these reports were accurate, the 660-km discontinuity could not be attributed to the post-spinel transition. Consequently, ringwoodite and bridgmanite + ferropericlase would not be the dominant minerals in the transition zone and lower mantle, respectively, which is highly unlikely. Therefore, the discrepancy between the reported results and expected pressures demands critical experimental investigations.

## High *P-T in situ* X-ray diffraction experiments by novel sophisticated techniques

In this study, we revisited the post-spinel transition pressure in Mg_2_SiO_4_ at 1700 K using a combination of advanced experimental LVP techniques and *in situ* X-ray diffraction. Details of the experimental procedure are shown in the Methods section. Here, we explain our strategies to determine the transition pressure. We paid close attention to controlling the sample pressure at the target temperature of 1700 K. Because the sample pressure decreases during the collection of an X-ray diffraction pattern upon heating, as discussed in refs^4,9^, we increased the press load to maintain a target pressure at 1700 K (the forced-pumping technique^10^). The starting material was a mixture of Mg_2_SiO_4_ forsterite, MgSiO_3_ enstatite and MgO periclase, allowing for both normal (ringwoodite → bridgmanite + periclase) and reversal (bridgmanite + periclase → ringwoodite) reactions. We determined the transition pressure with a typical pressure precision of ~0.05 GPa (Supplementary Fig. 1), which is one order of magnitude better than that of previous studies (~0.2 GPa)^2–5^, by collecting high-count and clean diffraction patterns of the MgO pressure marker^10^. Such high precision removes ambiguity when comparing the transition pressure to the discontinuity depth. Our preliminary experiments showed that pressure precision and control become worse at higher temperatures. This effect is due to a reduction in the quality of the MgO diffraction patterns derived from the disappearance of the MgO peaks via grain growth and the larger and irreproducible pressure drop across runs. Although the mantle temperature is estimated to be 2000 K at the 660-km discontinuity, we found that an experiment with a constant pressure was not feasible at this temperature. We therefore conducted the experiment at a temperature of 1700 K to determine the post-spinel transition pressure in this study.

## New results of the post-spinel transition pressure in complete agreement with the discontinuity depth

**Figure 1:**
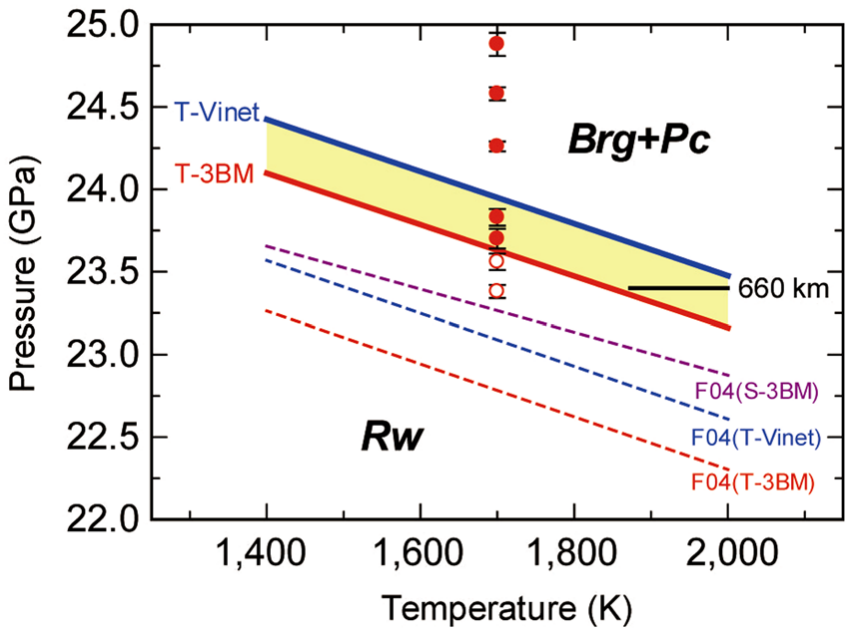
Phase boundaries of the post-spinel transition in Mg_2_SiO_4_. Data points were calculated based on the Tange 3BM MgO scale^11^. Open and solid circles with error bars identify the stable phases as ringwoodite and bridgmanite + periclase, respectively. The black solid line is the expected condition of the 660-km discontinuity^7,8,16–21^. The red (T-3BM) and blue (T-Vinet) solid lines are phase boundaries evaluated using the fixed points obtained in this study and the Clapeyron slopes of ref.^4^ after re-calculation with the Tange 3BM and Vinet MgO scales, respectively. The purple dashed line [F04 (S-3BM)] is the original phase boundary determined by ref.^4^ using the MgO scale proposed by ref.^12^. The aforementioned red [F04 (T-3BM)] and blue [F04 (T-Vinet)] dashed lines are therefore the phase boundaries recalculated from the original data of ref.^4^ using the specified scales. Brg: bridgmanite; Pc: periclase; Rw: ringwoodite.

We performed seven runs to determine the transition pressure at 1700 K (Table 1). The post-spinel transition pressure at 1700 K was constrained to be between 23.51 GPa and 23.76 GPa (Fig. 1) and between 23.83 GPa and 24.07 GPa (Table 1) according to the MgO scales based on the third-order Birch–Murnaghan and Vinet equations of states, respectively, reported by ref.^11^ (for details concerning the pressure scales, see the Methods section). These scales, hereafter referred to as the Tange 3BM and Vinet MgO scales, respectively, are currently the most accurate scales available because they were constructed based on pressure-scale-free datasets. Even though ref.^4^ reported a transition pressure of 23.3 GPa at 1700 K based on the MgO pressure scale proposed by ref.^12^, recalculation using the Tange 3BM and Vinet MgO scales provides transition pressures of 22.8 GPa and 23.1 GPa, respectively, which are 1 GPa lower than the present results (Fig. 1). Other studies using similar techniques with Au scales^2,3,5^ have likewise reported 1–2 GPa lower pressures.

**Table 1:** Experimental conditions and phases present at 1700 K.

Run no.	*P*_3BM_ (GPa)^a^	*P*_Vinet_ (GPa)^b^	*D*_1700 K_ (min)^c^	Phases
M2271	23.38(4)	23.69(4)	90	Rw + (Brg + Pc)^d^
M2272	23.56(5)	23.88(5)	60	Rw + (Brg + Pc)^d^
M2268	23.70(6)	24.02(6)	100	Brg + Pc
M1991	23.83(5)	24.16(5)	120	Brg + Pc
M2258	24.26(3)	24.59(3)	70	Brg + Pc
M2263	24.58(4)	24.91(5)	80	Brg + Pc
M2277	24.88(7)	25.22(7)	30	Brg + Pc

Abbreviations: Rw, ringwoodite; Brg, bridgmanite; Pc, periclase.

^a,b^Pressures calculated from the third-order Birch–Murnaghan and the Vinet equations of states of MgO by ref.^11^, respectively.

^c^Duration for which the temperature was maintained at 1700 K.

^d^These were judged to be metastable phases (see the Methods section).

The numbers in parentheses refer to the standard deviation in the last digit of the preceding number.

Here, we examine the correspondence of the post-spinel transition with the 660-km discontinuity using the new transition pressures obtained in this study. The temperature at the 660-km discontinuity is estimated to be 1900–2000 K^7,8^. Because we determined the transition pressures at 1700 K due to the technical reasons on determining the post-spinel transition pressure satisfied the present requirements as mentioned above, the transition pressure was extrapolated to 2000 K using the Clapeyron slope of ref.^4^ corrected by the Tange MgO scale (−0.0016 GPa/K), which gave 23.2 GPa and 23.5 GPa at 2000 K based on the Tange 3BM and Vinet MgO scales, respectively (Fig. 1). Taking into account the uncertainty of the Clapeyron slope between −0.003 GPa/K and −0.001 GPa/K^1–5,13–15^, the transition pressure would be 22.7–23.3 GPa and 23.4–23.8 GPa, respectively, according to these scales. Meanwhile, the global average depth of the discontinuity is 660 ± 10 km, a variance in distance that corresponds to a pressure variance of 23.4 ± 0.4 GPa^16–21^. Given these facts, the present findings are entirely consistent with the depth of the 660-km discontinuity, therefore corroborating the compositional models in which the Earth’s mantle is composed of ferromagnesian silicates.

Because the Earth’s mantle is a multicomponent system, additional components in ringwoodite and bridgmanite could affect the transition pressure. Ferrous iron (FeO), which is substituted for 10 mol% of MgO in ringwoodite, is one of the most important of such components. However, a previous study^1^, in which a topological relationship of the Mg_2_SiO_4_-Fe_2_SiO_4_ binary post-spinel transition loop was determined, demonstrated that the transition pressure changes at 1373 and 1873 K were less than 0.1 GPa at the composition of (Mg_0.9_,Fe_0.1_)_2_SiO_4_, implying that this substitution has only a limited effect on the transition pressure. The Earth’s lower mantle is considered to be under very reduced conditions, below the Fe-FeO buffer^22^. Reference^23^ showed that ferric irons in ringwoodite and ferropericlase are negligible when the redox state is at the Fe-FeO buffer and the Fe/(Fe + Mg) ratio in their minerals is 0.4. Reference^24^ showed that ferric iron in Al-free bridgmanite is negligible if it is under reduced conditions as in diamond capsules. For these reasons, the effects of ferric iron on the post-spinel transition pressure should be negligible in the Earth’s mantle. Another component that could affect the transition pressure is water. Because water is significantly incorporated in ringwoodite but not in bridgmanite and ferropericlase, water incorporation could increase the transition pressure. Reference^5^ reported that the transition pressure increases up to 1.5 GPa for 2% water at a temperature lower than 1700 K. However, because mantle temperatures, at least in the ambient mantle, are near 2000 K, the effects of water on the transition pressure are negligible in the majority of the mantle. Therefore, even when the effects of other components are considered, the depth of the 660-km discontinuity is definitely interpreted as the post-spinel transition in Mg_2_SiO_4_. The present study validates the applicability of widely accepted mantle compositional models such as the pyrolite and CI chondrite models.

Pressure scales such as equations of state of Au and MgO could contain uncertainties depending on the experimental data used to construct the equations of state. As mentioned above, previous studies have shown pressures that are 0.5–2 GPa lower than the discontinuity pressure, using the equations of state of Au and MgO, in which the MgO scales, including the Tange MgO scale, have given pressures that are 0.5–1 GPa lower. Although the Tange MgO scale used in this study does not guarantee accuracy for pressure, we emphasize that we obtained the highest transition pressure of all studies and complete agreement with the 660-km discontinuity. Temperature uncertainties derived from pressure effects on thermocouple electromotive force (emf) might affect the calculated pressures, although pressure effects on W-Re thermocouple have not yet been calibrated. Reference^25^ determined the absolute pressure effect on K-type thermocouple emf up to 7 GPa and 873 K, showing that the temperature correction is only 0 to −3 K. Therefore, the pressure effects would likely also be negligible for pressure determination in the case of W-Re thermocouple.

## Necessity of reinvestigation of phase relations of mantle minerals previously obtained by high *P-T in situ* X-ray diffraction

Previous studies likely underestimated the transition pressure owing to insufficient pressure control at high temperatures. The phase transition is completed within minutes after the target temperature is reached (see the Methods section). Reference^4^ reported a typical pressure decrease of ~0.5 GPa at a constant temperature of 1873 K; however, having adopted the final pressure before quenching in order to constrain the phase boundary, the authors significantly underestimated the transition pressure. We avoided a pressure drop at high temperature via forced pumping and, therefore, obtained a more accurate transition pressure. For comparison, pressure–time relations with and without forced pumping are shown in Supplementary Fig. 2. The pressure difference between 1100 K and 1700 K with forced pumping is close to zero (~0.2 GPa), whereas without forced pumping at 1700 K the pressure decreases by 1 GPa by the end of the first measurement. Therefore, the forced-pumping technique is essential to determine a phase boundary. In addition, we found that pre-heating suppressed decreases in the pressure at the target temperature (see the Methods section) that occurred even with forced pumping. We chose a pre-heating temperature of 1100 K so that the target phase transitions would not occur as a consequence of sluggish kinetics. Notably, we observed a lower transition pressure (22.8 GPa) in preliminary experiments without these techniques.

The problem of pressure drops is obvious when LVP is combined with *in situ* X-ray diffraction at high temperatures, even though it is in fact attributable to the high precision of the LVP experiments. Experiments in a laser-heated diamond anvil cell would therefore encounter similar difficulties. The only likely exception is the binary phase boundary of the olivine–wadsleyite transition determined by our co-author (ref.^10^), who also suppressed the drop in pressure using forced pumping. Astonishingly, this issue has never been seriously considered when investigating phase relations at high pressures and high temperatures. Therefore, there is a pressing need to reinvestigate every high P–T phase boundary that has been determined by X-ray diffraction without considering this problem. In particular, the determination of mantle mineral phase boundaries that are inseparably linked to other seismic discontinuities, for example, the 520-km and 720-km discontinuities probably implicated with the wadsleyite-to-ringwoodite transition and completeness of the garnet-to-bridgmanite transition (e.g. refs^26,27^), respectively, should be carefully investigated to improve our knowledge of the structure of the mantle.

## Methods

### Starting material

The starting material included Mg_2_SiO_4_ (forsterite), MgSiO_3_ (enstatite) and reagent-grade MgO (periclase) mixed at a molar ratio of 1:1:1. The forsterite and enstatite were prepared by the sol-gel method. Mg metal was dissolved in pure water to which HNO_3_ was added, making a solution of Mg(NO_3_)_2_. Tetraethylorthosilicate ((CH_3_CH_2_O)_4_Si) was mixed with the Mg(NO_3_)_2_ solution in 2:1 and 1:1 molar ratios of Mg:Si for forsterite and enstatite, respectively. Ammonia was added to the solutions to make the gels, which subsequently underwent stepwise heating to 1700 K to obtain forsterite and enstatite with good crystallinity. This mixture was ground to grain sizes of 1–3 μm and was sintered under 2 GPa of pressure at 800 K for 1 hr to form disks that were 1.2 mm in diameter and 0.5 mm thick. The pressure marker was reagent-grade MgO, with no additional material. It was sintered under the same conditions as the sample and made into disks of the same dimensions. These disks were cut into halves or quarters.

### High-pressure and high-temperature experiments combined with *in situ* X-ray diffraction and pressure determination

High-pressure and high-temperature experiments were conducted using SPEED-*Mk.II*, the LVP installed in beamline BL04B1 at the Japanese synchrotron radiation facility SPring-8^28^. The inner anvils were made of tungsten carbide (WC), grade TF05, produced by Fuji Die Co. Ltd. The anvil truncations for compressing the pressure medium were 4.0 mm. The anvil faces around the truncation were tapered by 1° to create a wide vertical opening to accommodate the X-rays^29,30^. Supplementary Fig. 3 shows a cross section of the cell assembly. The pressure media consisted of Cr_2_O_3_-doped, semi-sintered MgO octahedra with 10-mm edge lengths. Half-disks of the sample and pressure marker were surrounded by a 50-μm Mo foil and then placed in a MgO sleeve at the centre of a LaCrO_3_ cylindrical heater positioned parallel to the incident X-rays. Ta electrodes placed at both ends of the heater created an electrical connection with the WC anvils. A ZrO_2_ sleeve was placed outside the heater. Diamond/epoxy rods were positioned at both ends of the sample, and boron/epoxy rods were positioned at both ends of the diamond/epoxy rods. Two other boron/epoxy rods were placed in grooves of pyrophyllite gaskets in the X-ray path. The diamond/epoxy and boron/epoxy rods suppressed X-ray absorption, and the former, owing to their very low compressibility, prevented the X-ray path in the heater from closing under high P–T conditions. A W_97_Re_3_-W_75_Re_25_ thermocouple was inserted into the cylindrical heater normal to the axis that touched the Mo foil to monitor sample temperature. The thermocouple was electrically insulated from the heater using alumina tubes. The diffraction patterns of the MgO pressure marker were taken from the region next to the thermocouple (a couple hundred micrometers away from the thermocouple), which showed no systematic difference in pressure. Reference^3^ measured the temperature variation of a sample in the direction parallel to an incident X-ray using a cell assembly with nearly the same geometry as ours; those authors used a 1-mm-long sample and estimated the variation at 20 K. Because the sample length in our study was only 0.5 mm, the temperature variation would have been less than 20 K.

Energy-dispersive X-ray diffraction was conducted using white X-rays from a bending magnet. The X-ray beam was typically collimated to 50 μm horizontally and 100–700 μm vertically. The vertical dimensions were adapted to suit the sample conditions. The diffraction angles (2*θ*) were fixed to 7.2° and corrected before compression, using a sintered MgO sample and the unit-cell parameter of MgO at ambient conditions (*a*_0_ = 4.2112 Å). The relative error in the unit-cell parameter was 10^−4^, whereas that in the diffraction angle was 10^−5^. Diffracted X-rays were collected for 150–300 s using a germanium solid-state detector with an energy range extending to approximately 160 keV. Channel-energy calibration of the detector was conducted using the energies of the X-ray emission line (Kα) of ^55^Fe and γ radiation from ^57^Co and ^133^Ba. Samples were oscillated around the vertical axis between 0° and 7.2° to suppress the effects of grain growth^28^. Unit-cell volumes of the MgO pressure markers were typically calculated from eight diffraction peaks (111, 200, 220, 311, 222, 400, 420 and 422).

Generated pressures were determined based on the unit-cell volume of MgO obtained by X-ray diffraction, with temperatures indicated by a thermocouple using the MgO pressure scales that Reference^11^ developed from the third-order Birch–Murnaghan and Vinet equations of state (EOSs). The pressures measured just after the temperatures reached 1700 K were adopted as the run pressures. Pressures calculated using these EOSs should be the most accurate and reliable of the proposed MgO scales because they were based on experimental data without the use of any other pressure scale, such as thermal expansivity at elevated temperatures and ambient pressure, the adiabatic bulk modulus at high temperatures and ambient pressure, relations between the volume and the adiabatic bulk modulus under compression at ambient temperature, and shock compression. The high degree of precision for the typical pressure determination, 0.05 GPa, was achieved by using multiple (usually eight) diffraction peaks of MgO in “clean” diffraction patterns without additional peaks, except for the weak ones from the diamond in front of and behind the sample in the X-ray path. In general, use of peaks on the high-energy side enhances the precision of a unit-cell parameter due to their more precise *d*-values compared with peaks on the low-energy side. In fact, pressure precision became worse (±0.1–0.2 GPa) when we obtained the diffraction patterns with many impurity peaks and ambiguous high-energy peaks. In addition, the wide vertical opening created by the tapered anvils allowed a rapid counting rate and, therefore, contributed to the high degree of precision.

### Experimental procedure for the forced-pumping technique

A high-pressure assembly was compressed to a desired press load (6–7 MN) at ambient temperature [Supplementary Fig. 4a(I)], and the sample temperature was then increased to 1100 K [Supplementary Fig. 4a(II)]. This temperature was maintained for 30–90 min while forsterite converted to ringwoodite and enstatite converted to akimotoite, during which time stress in the sample assembly was relieved. Also during this time, the pressure on the samples gradually decreased by ~1 GPa [Supplementary Fig. 4a(III)], and the press load was then increased by 0.5–0.8 MN to bring the pressure to ~23 GPa [Supplementary Fig. 4a(IV)]. The temperature of the samples was next increased to 1700 K within 5 min, after which the press load was increased rapidly (~0.05 MN/min) for 5 min and then at a slower rate (0.01–0.02 MN/min) to suppress variations in the pressure on the samples (the forced-pumping technique). The energy-dispersive X-ray diffraction patterns of the pressure marker and sample were collected alternately to monitor the pressure variation and the reaction progress [Supplementary Fig. 4a(V)]. The increasing press load was adjusted based on previous pressure variations while holding the temperature steady. After remaining at the target temperature for 0.5–2 hr, the samples were quenched by cutting the electric power to the heater and allowed to decompress slowly; even so, avoiding blowout proved impossible. Because the akimotoite-to-bridgmanite transition is kinetically faster than the ringwoodite formation from bridgmanite + periclase^2,3,31,32^, the mineral assemblage before the reverse post-spinel transition should be ringwoodite + bridgmanite + periclase, causing the reaction not from akimotoite + periclase but from bridgmanite + periclase to ringwoodite. Therefore, we successfully conducted both the normal and reversal experiments at 1700 K in this run procedure.

### Determination of the stable assemblage

Stable assemblages in the present study were determined based on *in situ* X-ray diffraction patterns. Supplementary Figure 4b illustrates representative *in situ* X-ray diffraction patterns of samples within 10 min after reaching 1700 K. When the pressure was below 23.6 GPa, ringwoodite appeared along with small amounts of bridgmanite and periclase (Supplementary Fig. 4b lower); above this pressure, only bridgmanite and periclase appeared (Supplementary Fig. 4b upper). Diffraction patterns in each run remained unchanged while the target temperature was kept constant; therefore, the reactions ended within 10 min after reaching 1700 K.

We conclude that the runs with ringwoodite and small amounts of bridgmanite + periclase were within that mineral’s stability field (Supplementary Fig. 5). Each phase of bridgmanite and periclase was itself stable at the pressures investigated^4,31,33^, and contiguous bridgmanite and periclase grains formed ringwoodite only around the grain boundary region in the reversal reaction because of limited diffusivities of the constituent elements, resulting in bridgmanite and periclase grains remaining in the sample. This separation of the bridgmanite and periclase grains impeded completion of the ringwoodite formation. Conversely, no ringwoodite exists in the stability field of bridgmanite + periclase because it is unstable above the transition pressure and has a high decomposition rate^3^.

### Analysis of the recovered samples

Texture of the recovered samples was observed using a Zeiss LEO 1530 Gemini field-emission-type scanning electron microscope with an Oxford X-Max^N^ energy-dispersive X-ray spectrometer. The phases present in the recovered samples were confirmed using an in-house micro-focused X-ray diffractometer (Brucker AXS Discover 8).

### Data availability

Data in the manuscript, or any additional data can be requested by emailing the corresponding author Takayuki Ishii at takayuki.ishii@uni-bayreuth.de.

## Electronic supplementary material

Supplementary information
