# Supplementary material for: Complete agreement of the post-spinel transition with the 660-km seismic discontinuity
Source: Sci Rep. 2018 Apr 20;8:6358. doi: 10.1038/s41598-018-24832-y (PMC5910398; doi:10.1038/s41598-018-24832-y)
Supplement: Supplementary file 1 — Supplementary information [file 41598_2018_24832_MOESM1_ESM.docx]

Supplementary information for

**Complete agreement of the post-spinel transition with the 660-km seismic discontinuity**

**Authors:** Takayuki Ishii^*,1^, Rong Huang^1^, Hongzhan Fei^1^, Iuliia Koemets^1^, Zhaodong Liu^1^, Fumiya Maeda^2^, Liang Yuan^2^, Lin Wang^1^, Dmitry Druzhbin^1^, Takafumi Yamamoto^3^, Shrikant Bhat^1,4^, Robert Farla^4^, Takaaki Kawazoe^1^, Noriyoshi Tsujino^5^, Eleonora Kulik^1,4^, Yuji Higo^6^, Yoshinori Tange^6^, Tomoo Katsura^1^

**Affiliations:**

^1^Bayerisches Geoinstitut, University of Bayreuth, 95440 Bayreuth, Germany

^2^Department of Earth Sciences, Graduate School of Science, Tohoku University, Sendai, 980-0845, Japan

^3^Department of Earth and Planetary Systems Sciences, Graduate School of Science, Hiroshima University, Kagamiyama 1-3-1, Higashi-Hiroshima, 739-8526 Japan

^4^Deutsche Elektronen-Synchrotron (DESY), Notkestraße 85, 22607 Hamburg, Germany

^5^Institute for Study of the Earth’s Interior, Okayama University, Misasa, 682-0193 Japan

^6^Japan Synchrotron Radiation Research Institute (JASRI), 1-1-1, Kouto, Sayo-cho, Sayo-gun, Hyogo 679-5198 Japan

**^*^Corresponding author:** [takayuki.ishii@uni-bayreuth.de](mailto:takayuki.ishii@uni-bayreuth.de)


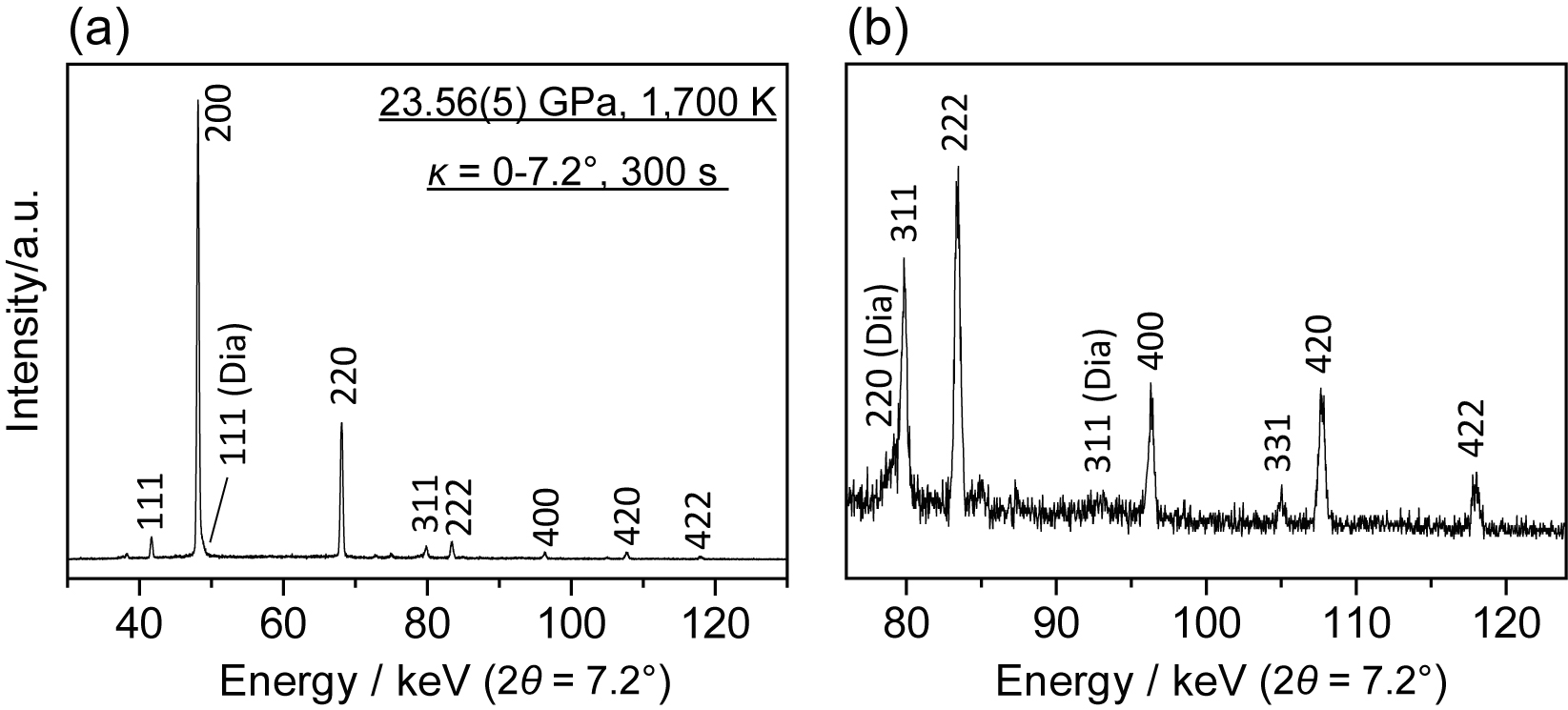


Supplementary Figure 1. The X-ray diffraction profile of an MgO pressure marker (run M2272) at the target temperature of 1700 K. (a) The X-ray diffraction pattern obtained just after reaching 1700 K. (b) An enlarged profile of the high-energy side (76–124 keV) of (a). Eight diffraction peaks of MgO (indices of 111, 200, 220, 311, 222, 400, 420 and 422) were typically used to calculate pressures. Only tiny diamond peaks, which were derived from diamond/epoxy placed at both sides of the samples, were observed as a secondary phase in the profile. *κ*: oscillation angle.


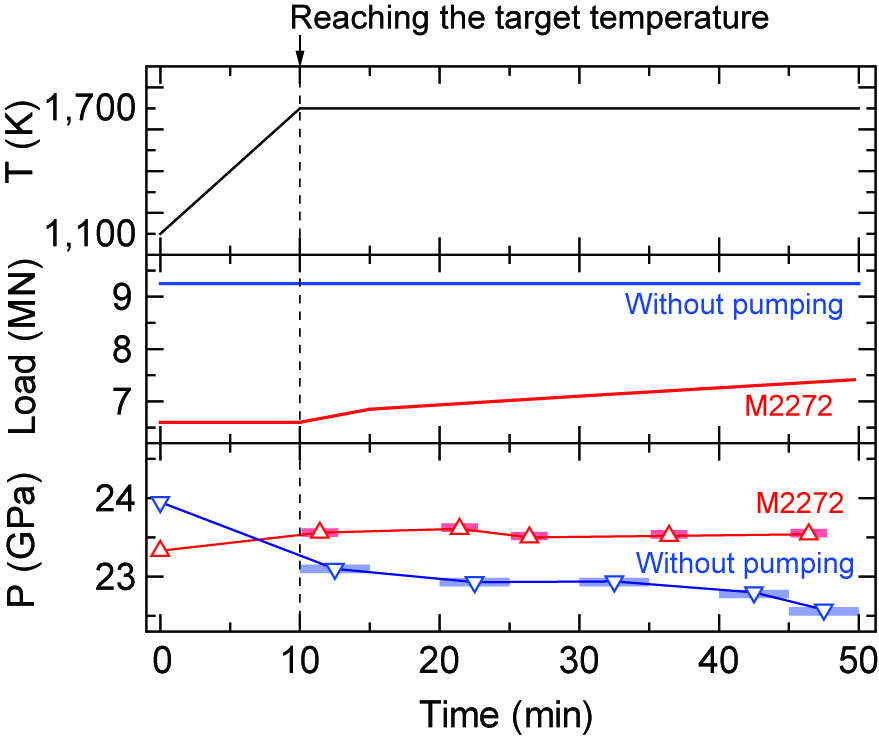


Supplementary Figure 2. Time dependence of pressure with the temperature and press load. The red lines and plots represent data for M2272 with forced pumping. The blue lines and plots represent a typical run without forced pumping. The red and blue bars in the plots indicate collection periods of X-ray diffraction patterns of MgO (150 and 300 s, respectively, in these runs), implying that the pressures plotted were average values during collection. In the run without forced pumping, where bridgmanite + periclase was observed, the pressure at 1100 K dramatically decreased by the end of the first pressure measurement just after reaching 1700 K (~0.9 GPa). Conversely, in M2272, the pressure difference was ~0.2 GPa. The errors in the pressures are smaller than the plot size.


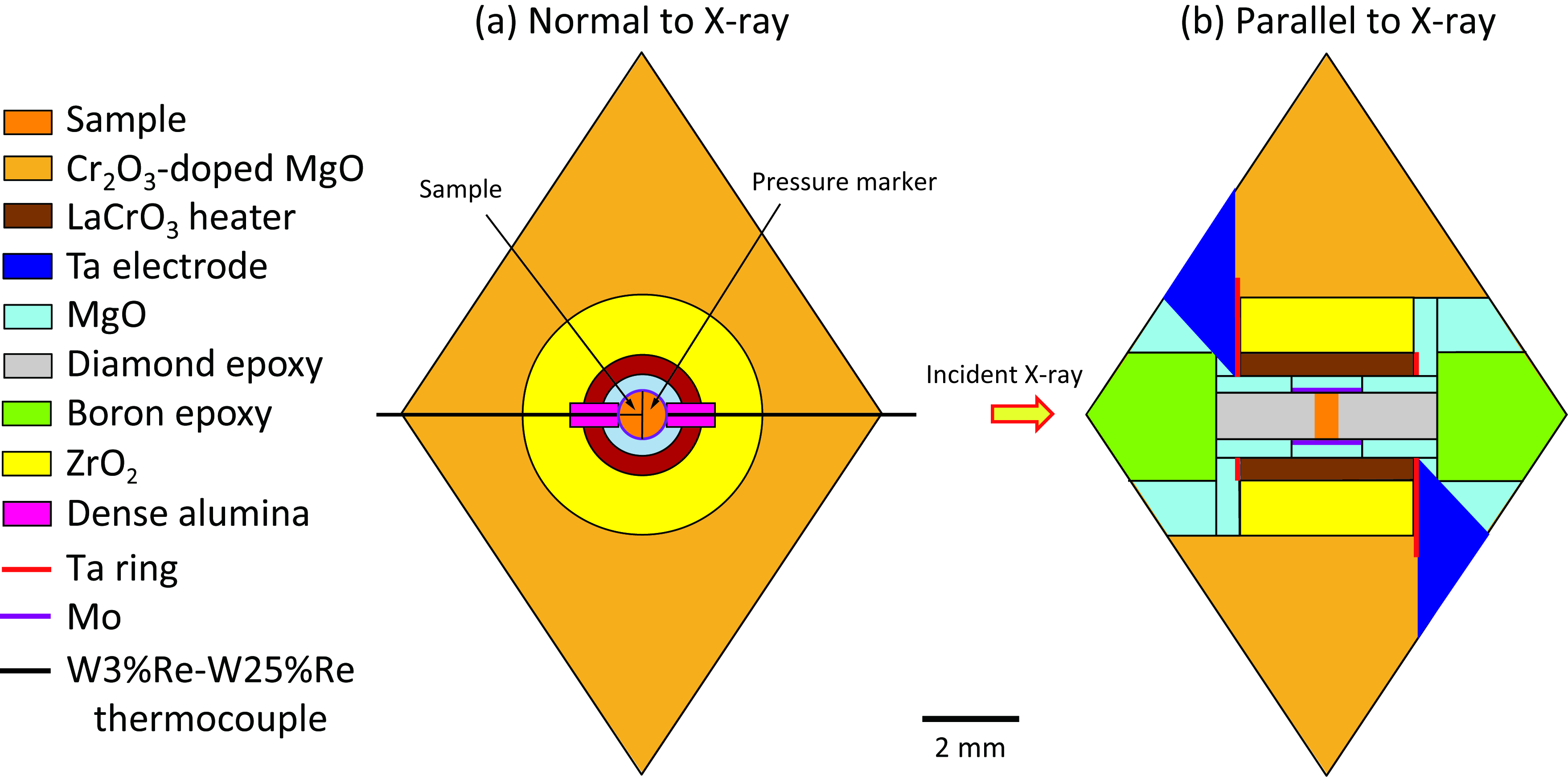


Supplementary Figure 3. Schematic cross section of a cell assembly.


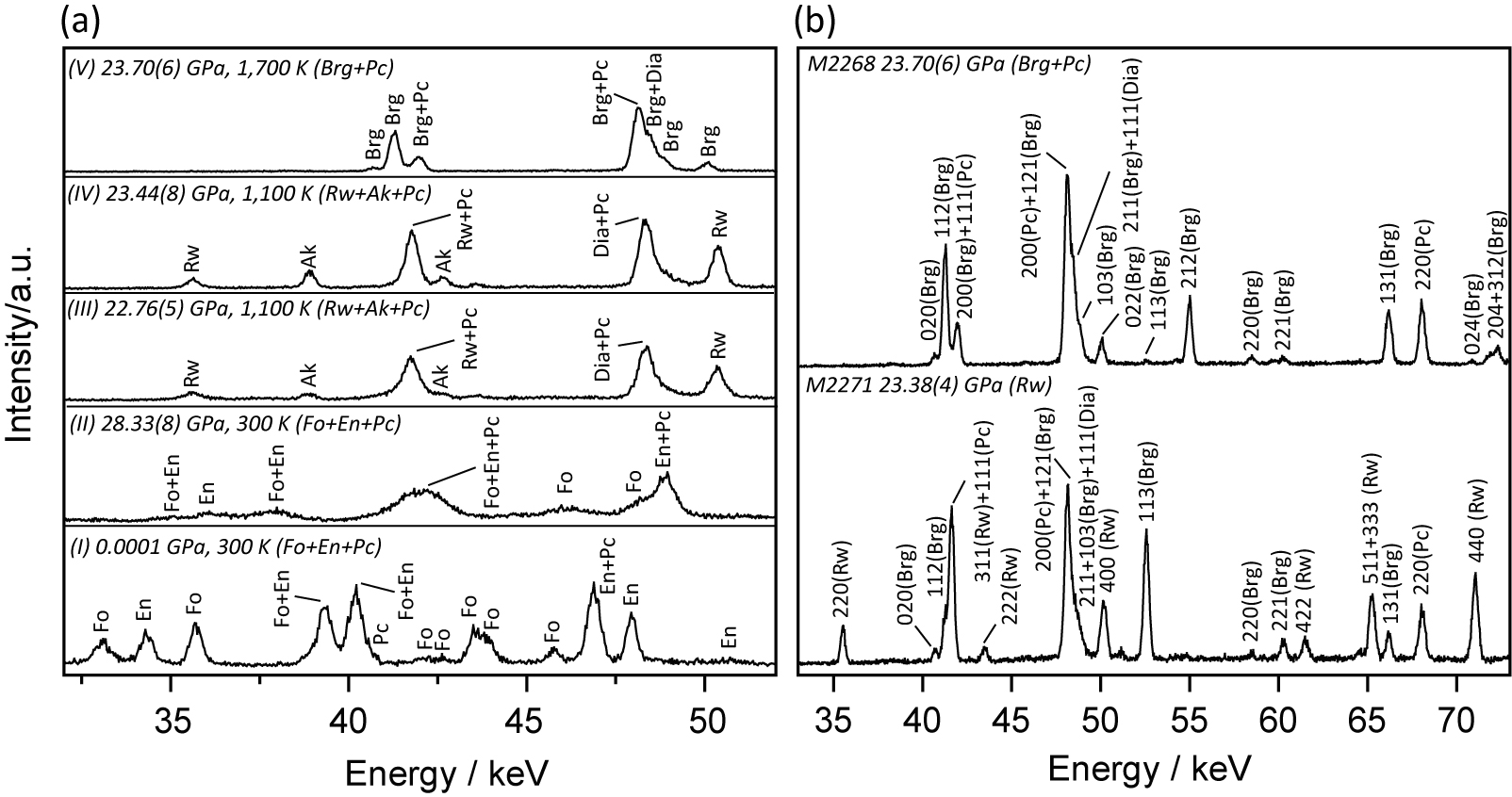


Supplementary Figure 4. Representative X-ray diffraction patterns in the present experiments. (a) Typical X-ray diffraction pattern changes in the present compression and heating processes (M2268). The starting sample was compressed to 28.33(8) GPa at room temperature (II) and then heated to 1100 K to synthesize an assemblage of ringwoodite + akimotoite + periclase with a pressure decrease to 22.76(5) GPa (III). While the temperature was kept constant, the press load was increased to obtain a pressure of ~23 GPa, for a resulting pressure of 23.44(8) GPa (IV). Next, the temperature was increased to 1700 K and each X-ray diffraction measurement of the pressure marker and sample was conducted within 10 min (V). (b) X-ray diffraction patterns obtained at pressures of 23.38(4) GPa (lower) and 23.70(6) GPa (upper) at a temperature of 1700 K. The mineral assemblages in the titles are the stable phases suggested by the presence or absence of ringwoodite. 2*θ* is ~7.2°. Fo: forsterite; En: enstatite; Rw: ringwoodite; Ak: akimotoite; Brg: bridgmanite; Pc: periclase; Dia: diamond/epoxy (see Supplementary Fig. 3).


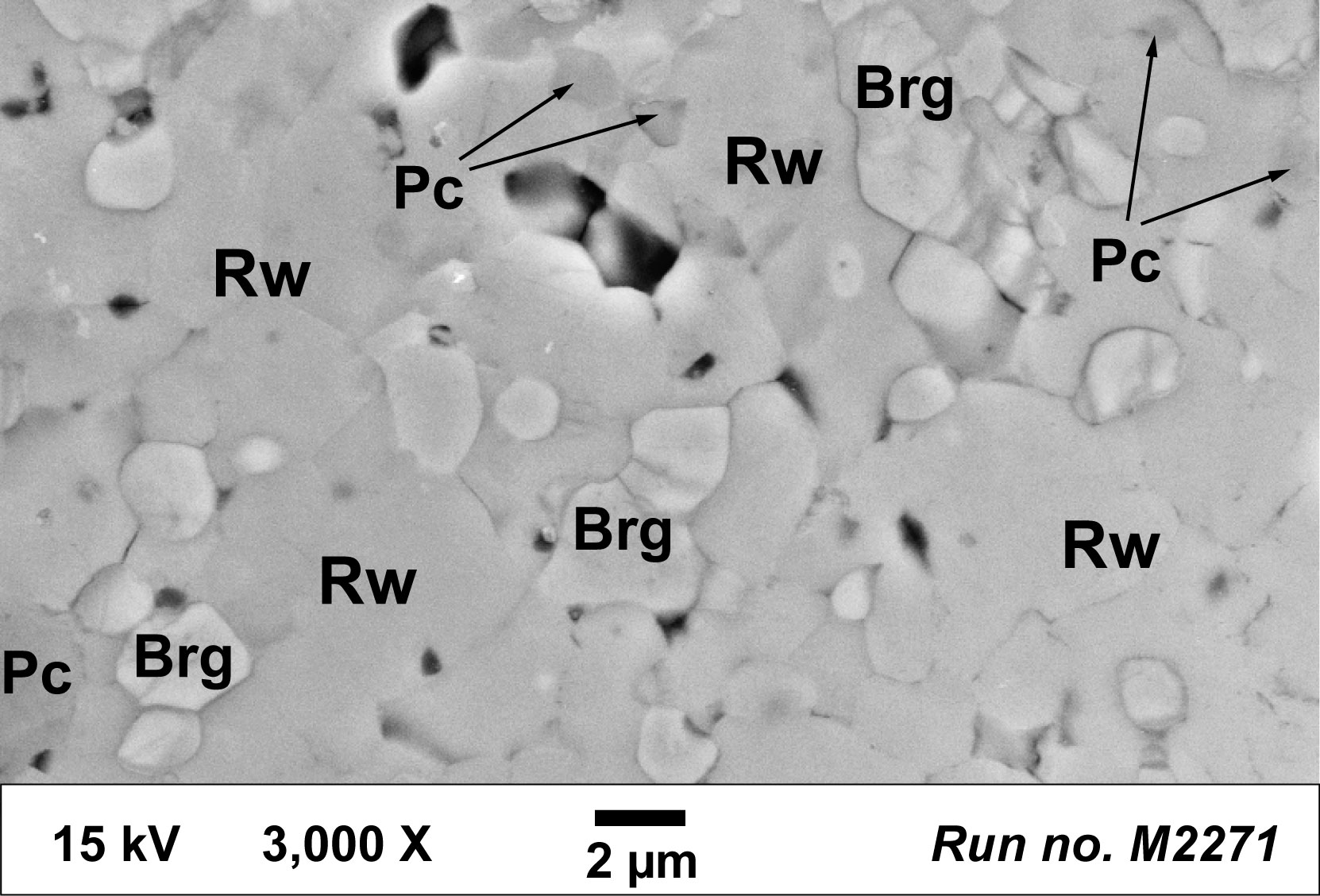


Supplementary Figure 5. A back-scattered electron image of the sample recovered from 23.38 GPa and 1700 K (M2271). The white, light-grey and dark-grey grains are bridgmanite, ringwoodite and periclase, respectively. Typical grains of bridgmanite and periclase measure 1–2 μm, comparable to those in the starting material. The bridgmanite and periclase grains are surrounded by bulk ringwoodite grains (2–6 μm), which separate each other and impede the complete reversal reaction between bridgmanite and periclase. Brg: bridgmanite; Pc: periclase; Rw: ringwoodite.
